# Protective effect and related mechanisms of curcumin in rat experimental periodontitis

**DOI:** 10.1186/s13005-018-0169-1

**Published:** 2018-08-16

**Authors:** Chang-Jie Xiao, Xi-Jiao Yu, Jian-Li Xie, Shuang Liu, Shu Li

**Affiliations:** 1 Shandong Provincial Key Laboratory of Oral tissue regeneration, Department of Periodontology, School and Hospital of Stomatology, Shandong University, 44-1# West Wenhua Road, Jinan, Shandong China; 2 Department of Endodontics, Jinan Stomatological Hospital, 101# Jingliu Road, Jinan, Shandong China

**Keywords:** Curcumin, NF-κB, OPG/RANKL, Periodontitis, Micro-CT

## Abstract

**Background:**

Curcumin exhibits anti-inflammatory effects and has been suggested as a treatment for inflammatory diseases. The aim of this study was to investigate the effects of curcumin on the lipopolysaccharide induced inflammatory response in rat gingival fibroblasts in vitro and ligation-induced experimental periodontitis in vivo, and to speculate the possible anti-inflammatory mechanism of curcumin.

**Methods:**

The gingival fibroblasts were incubated with different concentrations of curcumin in the absence or presence of lipopolysaccharide (LPS). Concentrations of interleukin-1β(IL-1β), tumor necrosis factor-α (TNF-α), osteoprotegerin (OPG) and soluble receptor activator of nuclear factor kappa-B ligand (RANKL) culture supernatants of rat gingival fibroblasts were determined by enzyme linked immunosorbent assay. The nuclear fraction of rat gingival fibroblasts was extracted and nuclear factor kappa-B (NF-κB) activation was assessed by western blotting to elucidate related mechanisms. Curcumin was given every two days by oral gavage. The gingival inflammation and alveolar bone loss between the first and second molars were observed by hematoxylin and eosin staining. Collagen fibers were observed by picro-sirius red staining. Alveolar bone loss was assessed by micro-CT analysis.

**Results:**

Curcumin attenuated the production of IL-1β and TNF-α in rat gingival fibroblasts stimulated by LPS, and inhibited the LPS-induced decrease in OPG/sRANKL ratio and NF-κB activation. Curcumin significantly reduced gingival inflammation and modulated collagen fiber and alveolar bone loss in vivo.

**Conclusions:**

curcumin modulates inflammatory activity in rat periodontitis by inhibiting NF-κB activation and decreasing the OPG/sRANKL ratio induced by LPS.

## Background

Periodontitis is a prevalent oral inflammatory disease characterized by progressive gingival tissue inflammation, irreversible alveolar bone loss and deep periodontal pockets. It is caused by accumulation of profuse amounts of dental plaque. The conventional treatment for periodontitis is to reduce dental bacteria levels by scaling and root planing [1]. Antibiotics such as doxycycline have been used to alter the host response to the periodontal pathogens by disrupting the action of matrix metalloproteinase and to thus minimize host-mediated tissue destruction [2], but systemic use of antibiotics can interfere with normal body systems and may cause several side effects,such as drug resistance [3].

Treatment of periodontitis in traditional Chinese medicine or natural substances is one of the research points in recent years. Several compounds extracted from spices and herbs exhibit anti-inflammatory effects, which suggest potential pharmacological uses. Curcumin, the principal curcuminoid in turmeric (*Curcuma longa*), has been used as a food additive and herbal supplement because of its potential medicinal properties [4]. Curcumin has been shown to exhibit anti-inflammatory biological activity [5–8]. Gingival tissues are the first tissues affected during the initial stage of periodontitis [9]. Gingival fibroblasts, as the major cell type in gingival tissues, which stimulated by lipopolysaccharide (LPS) can activate the nuclear factor kappa-B (NF-κB) signaling pathway and products inflammatory cytokines such as IL-1β and TNF-α. Extensive research has demonstrated that the transcription factor NF-κB is a key component of the inflammatory process [10]. However, the anti-inflammatory effects of curcumin on LPS-stimulated rat gingival fibroblasts and the molecular mechanisms remain unclear. The expression and activation of OPG and RANKL are crucial for alveolar bone absorption and metabolism [11]. The present study was undertaken to investigated the hypothesis that curcumin would inhibit the LPS-induced inflammatory response in rats gingival fibroblasts in vitro and ligation-induced experimental periodontitis in vivo.

## Methods

### Reagents

LPS and curcumin were purchased from Sigma (USA). NF-κB p-p65 and p-IκBα were purchased from Cell Signaling Technology (USA). IL-1β, TNF-α, OPG and soluble RANKL (sRANKL) ELISA kits were obtained from R & D Systems (Minneapolis, MN, USA). Wistar rats for the ligation-induced experimental periodontitis model were obtained from the Laboratory Animal Center of Shandong University (Shandong, China). This study was approved by the Local Ethics Committee of the Animal Care and Use Committee of the School of Stomatology, Shandong University.

### Cell culture

Normal gingival tissues were obtained from male Wistar rats (aged 5 weeks) that were clinically free of periodontal disease. Enzymatic digestion were adopted and maintained in Dulbecco’s modified Eagle’s medium (DMEM; Gibco, USA) containing 20% fetal bovine serum (FBS), 100 U/mL penicillin and 100 mg/mL streptomycin (Hyclone, Beijing, China). After reaching confluence, the cells were detached from the culture surface with 0.25% trypsin and subcultured in DMEM containing 10% FBS and antibiotic solution. The medium was changed every 48 h. Gingival fibroblasts between passages 4 and 7 were used in this study.

### Cell viability

The cell viability of gingival fibroblasts was assessed using the MTT assay as previously described [12]. Briefly, gingival fibroblasts were seeded in 96-well plates (1 × 10^4^ cells per well) and cultured for 12 h. The cells (LPS, LPS+ 10 μM curcumin, LPS+ 20 μM curcumin, 10 μM curcumin, 20 μM curcumin and normal fibroblasts as control)(*n* = 8) were incubated with different concentrations of curcumin in the absence or presence of LPS (1 μg/ml) [13, 14] for 24 h. Then, 20 μl of MTT (5 mg/ml) was added to each well and the cells were incubated for 4 h. The medium was then removed and 150 μl of DMSO was added to each well. Optical density was measured at 450 nm using a Bio-Rad microplate reader (model 680, Bio-Rad, USA).

### ELISA assay

The concentrations of IL-1β, TNF-α, OPG and sRANKL in the culture supernatants of gingival fibroblasts incubated with different concentrations of curcumin in the absence or presence of LPS (1 μg/ml) for 24 h were measured using commercially available ELISA kits [15, 16]. ELISA assays were performed according to the manufacturer’s instructions.

### Protein extraction and western blotting

The nuclear fraction of gingival fibroblasts was extracted for NF-κB evaluation using an Ambion PARIS system (Thermo Fisher). Protein concentrations were measured using a bicinchoninic acid quantitative protein analysis assay kit (Boshe, China). Proteins were separated on 10% SDS gels and transferred onto polyvinylidene difluoride membranes (Millipore, USA). After being blocked in 0.1% Tween 20 in Tris-buffered saline containing 5% nonfat dried milk for 1 h at room temperature, the membranes were incubated with NF-κB, p-p65 and p-IκBα (all diluted 1:1000) overnight at 4 °C. The membranes were then rinsed with TBST for 10 min three times, and incubated with horseradish peroxidase-labeled second antibody (Beyotime). Immunoreactive bands were visualized on Canon film using enhanced chemiluminescence substrate solution (Millipore). Histone H3 (antibody diluted 1:10000) was used as an internal control.

### Animals

Twenty-four male Wistar rats that had undergone this ligation procedure were randomly distributed into the following 3 groups: a ligation-only (L) group, a group treated with 30 μg/g body weight curcumin (L + C_30_), and a group treated with 100 μg/g body weight curcumin (L + C_100_). Curcumin diluted in corn oil vehicle was administered every 2 days by oral gavage, starting the day before ligation. Animals in the L group were administered the same volume of the corn oil vehicle. Food and water were provided ad libitum.

### Ligation-induced experimental periodontitis

The procedure used for ligation-induced experimental periodontitis was as previously described [17]. Briefly, a 4–0 silk suture and an orthodontic ligature wire were passed through the interdentium between the first and second molars using Dumont forceps, and then the silk suture was wound tightly around the orthodontic ligature wire to cover it. After the gingiva was lacerated by a dental probe, the orthodontic ligature wire was ligated firmly to the dental cervix of the right first lower molar.

### Micro-computerized tomography (micro-CT) analysis

SkyScan 1176 (BRUKER, USA) at 65 kV and 380 μA was applied for micro-CT analysis. Mandibles were scanned at 9-μm resolution. Three-dimensional (3D) volume viewing and analysis software (DataViewer, CT-volume and CT-analyser, SkyScan, Bruker, USA) were used to visualize and quantify 2D and 3D data on a personal computer output, and a standardized gray-scale value was used to visualize mineralized tissues only.

### Collagen fibers analysis

Sections were deparaffinized, hydrated and washed, then stained with 0.1% picro-sirius red for 60 min and rinsed with hydrochloric acid (0.01 M) for 2 min. The sections were dehydrated and sealed with mounting medium, and then the gingival fibers were analyzed under a polarizing microscope (Olympus BHSP, Japan).

### Statistical analysis

All data are presented as means ± SD of three independent experiments. Data were statistically analyzed by one-way analysis followed by the Newman–Keuls post hoc test using SPSS 17.0 statistical software (SAS, Cary, NC, USA). *P* < 0.05 was considered statistically significant.

## Results

### Effects of curcumin on cell viability

**Fig. 1:**
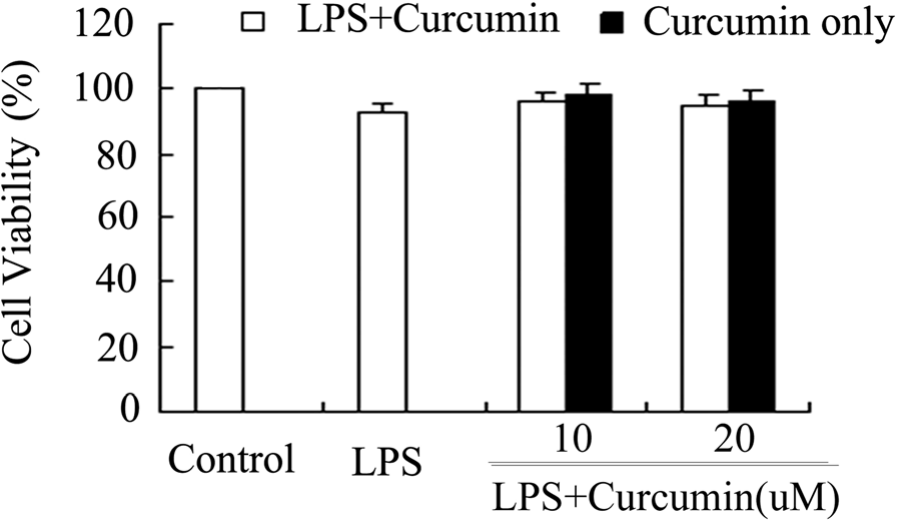
Effects of curcumin on the cell viability of rat gingival fibroblasts. Cells were cultured with different concentrations of curcumin (0, 10 and 20 μM) in the presence or absence of 1 μg/ml lipopolysaccharide (LPS). Cell viability was determined by MTT assay. Curcumin was not cytotoxic to gingival fibroblasts at 10 or 20 μM (*P* > 0.05)

The cytotoxic effect of curcumin on rat gingival fibroblasts was assessed by the MTT assay. There was no significant difference in cell viability in LPS + 10 μM curcumin-treated cells, LPS + 20 μM curcumin-treated cells, 10 μM curcumin-treated cells or 20 μM curcumin-treated cells compared with control fibroblasts (*P* > 0.05). These results show that 10 and 20 μM curcumin were not cytotoxic to gingival fibroblasts (Fig. 1). Thus, curcumin was used at 10 and 20 μM in the subsequent in vitro studies.

### Effects of curcumin on TNF-α and IL-1β expression in gingival fibroblasts

**Fig. 2:**
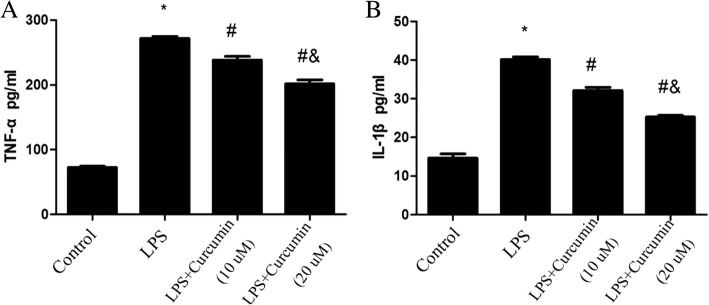
Effects of curcumin on the expressions of TNF-α and IL-1β in culture supernatants of rat gingival fibroblasts. Curcumin decreased the levels of TNF-α and IL-1β stimulated by LPS. *Different from Control group (*P* < 0.05). # Different from LPS group (*P* < 0.05). & Different from LPS+ Curcumin (10 μM) group (*P* < 0.05)

The culture supernatants of gingival fibroblasts treated with LPS, LPS + 10 μM curcumin, and LPS + 20 μM curcumin, and from the untreated control cells were tested. TNF-α and IL-1β in culture supernatants were markedly elevated in the LPS group comparing with the Control group (*P* < 0.05). 10 and 20 μM Curcumin decreased the levels of TNF-α and IL-1β stimulated by LPS in the culture supernatants of gingival fibroblasts (*P* < 0.05) (Fig. 2).

### Effects of curcumin on LPS-induced NF-κB activation in vitro

**Fig. 3:**
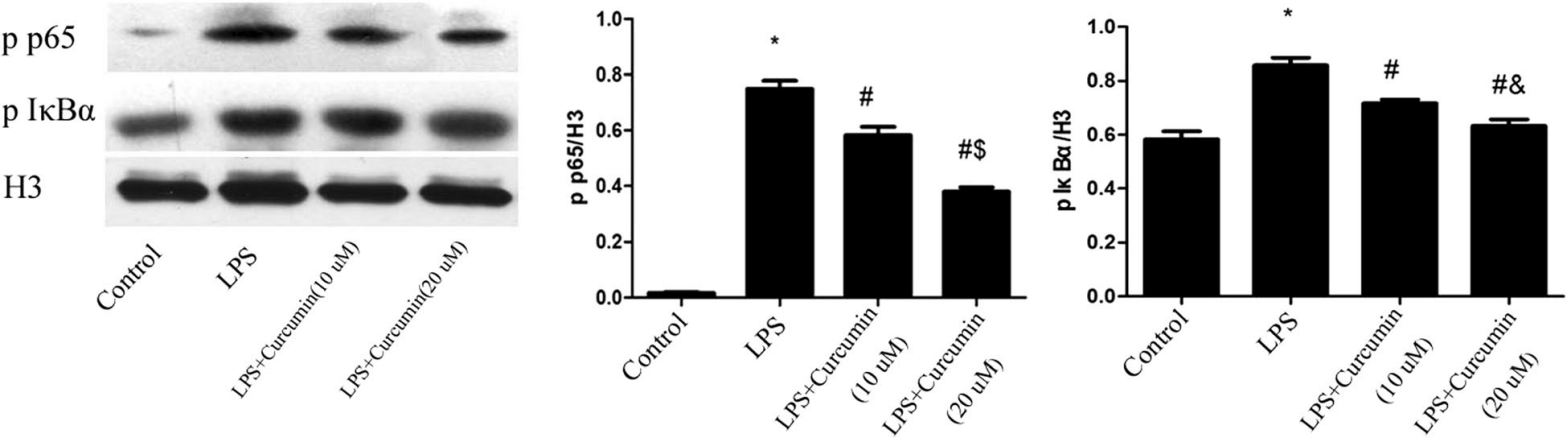
Effects of curcumin on LPS-induced NF-κB activation. Curcumin significantly inhibited NF-κB activation induced by LPS. *Different from Control group (*P* < 0.05). # Different from LPS group (*P* < 0.05). & Different from LPS+ Curcumin (10 μM) group (*P* < 0.05)

Cells were treated with curcumin (10, 20 μM) in the presence of LPS (1 μg/ml) for 24 h. Cell nuclear protein samples were analyzed by western blotting. The results showed that LPS significantly increased NF-κB, p-p65 and p-IκBα levels (*P* < 0.05). Moreover, curcumin significantly inhibited LPS-induced NF-κB activation (*P* < 0.05).*Different from Control group (*P* < 0.05). # Different from LPS group (*P* < 0.05). & Different from LPS+ Curcumin(10 μM) group (*P* < 0.05) (Fig. 3).

### Effects of curcumin on OPG/sRANKL ratio in gingival fibroblasts

**Fig. 4:**
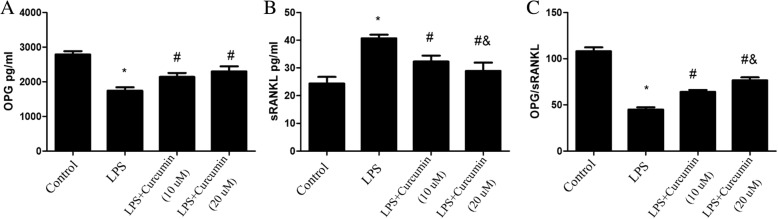
Effects of curcumin on OPG/sRANKL ratio in culture supernatants of rat gingival fibroblasts. LPS downregulated OPG release from gingival fibroblasts; Curcumin reduced the downregulation (**a)**. The sRANKL level in the culture supernatant of the LPS-treated cells was significantly higher than that of the control cells. LPS upregulated sRANKL release from gingival fibroblasts; curcumin reduced this upregulation (**b**). The OPG/sRANKL ratio decreased significantly in the LPS group. Curcumin inhibited the LPS-induced decrease in the OPG/sRANKL ratio (**c**). *Different from Control group (*P* < 0.05). # Different from LPS group (*P* < 0.05). & Different from LPS+ Curcumin (10 μM) group (*P* < 0.05)

The level of sRANKL in LPS-treated cells was significantly higher than that in control cells (*P* < 0.05). OPG release was significantly decreased when gingival fibroblasts were treated with LPS. Therefore, the OPG/sRANKL ratio decreased significantly in the LPS-treated cells. Curcumin reduced sRANKL release from gingival fibroblasts, and also increased OPG release. Thus, curcumin inhibited the LPS-induced decrease in the OPG/sRANKL ratio (*P* < 0.05) (Fig. 4).

### Effects of curcumin on ligation-induced experimental periodontitis in vivo

**Fig. 5:**
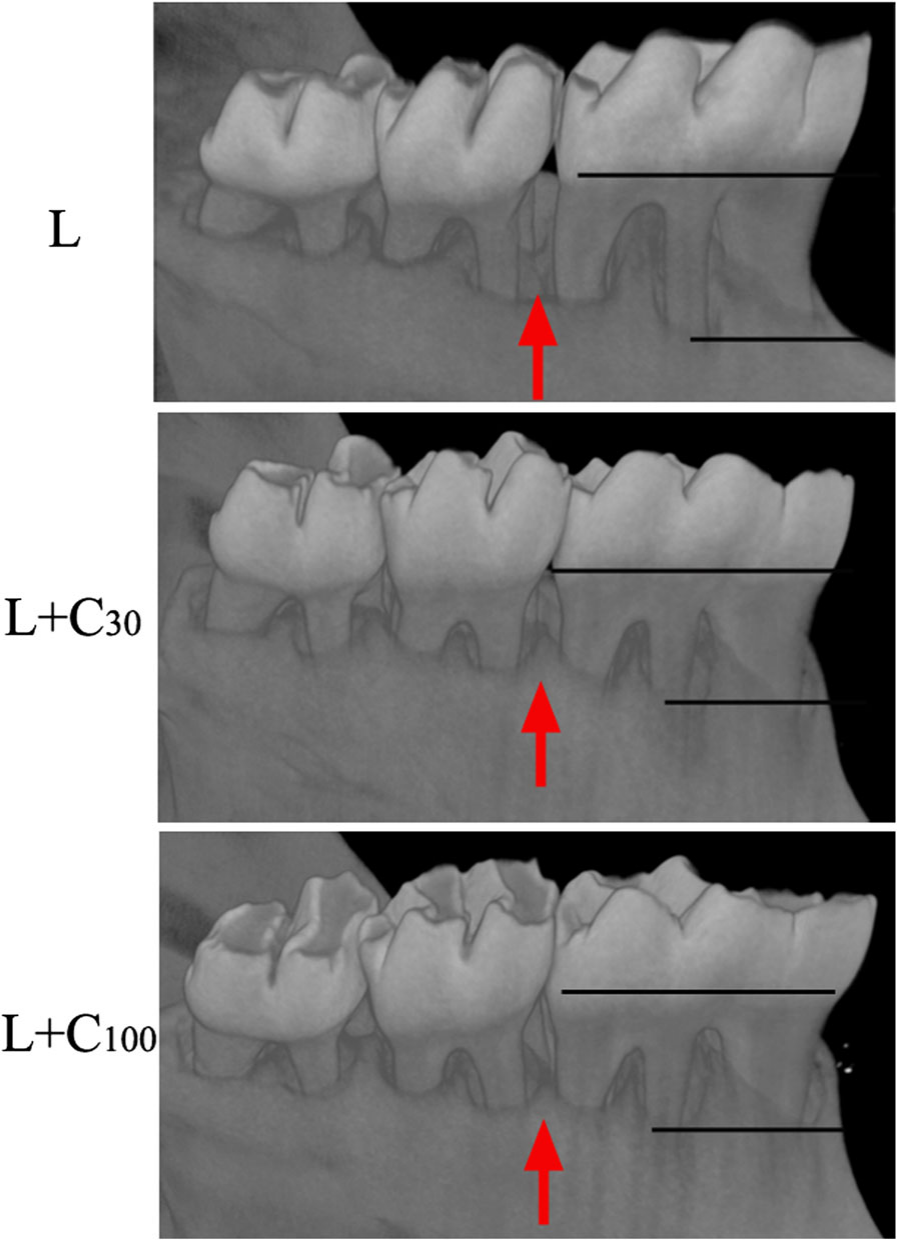
Alveolar bone loss in mandible by Micro-CT. Alveolar bone between the first and second molars was observed (red arrow). Alveolar bone height was obviously decreased in the L group. Alveolar bone loss was alleviated in the L + C_30_ and L + C_100_ groups

**Fig. 6:**
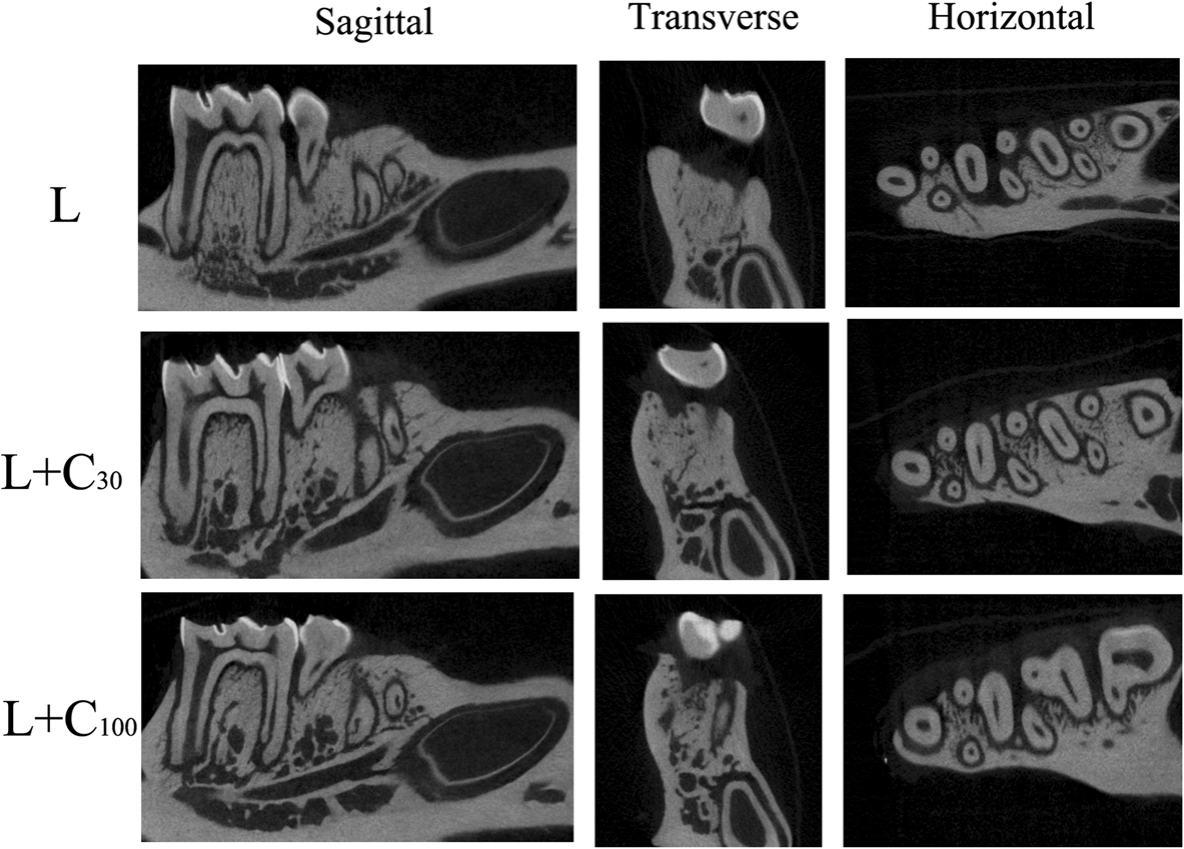
Three-dimensional images of alveolar bone loss in rat experimental periodontitis by Micro-CT. Alveolar bone loss was observed in all the three groups. Obvious alveolar bone loss between the first and second molars was observed in the L group. Alveolar bone loss was alleviated in the groups that underwent ligation in combination with treatment with 30 μg/g body weight curcumin (L + C_30_) or 100 μg/g body weight curcumin (L + C_100_)

Alveolar bone loss in the mandible between the first and second molars was observed by micro-CT (Fig. 5). Alveolar bone loss was observed in all three groups of rats. Alveolar bone crest height was obviously decreased, and notable alveolar bone loss between the first and second molars was observed in the L group (Fig. 6). Alveolar bone loss was reduced in the L + C_30_ and L + C_100_ groups.

**Fig. 7:**
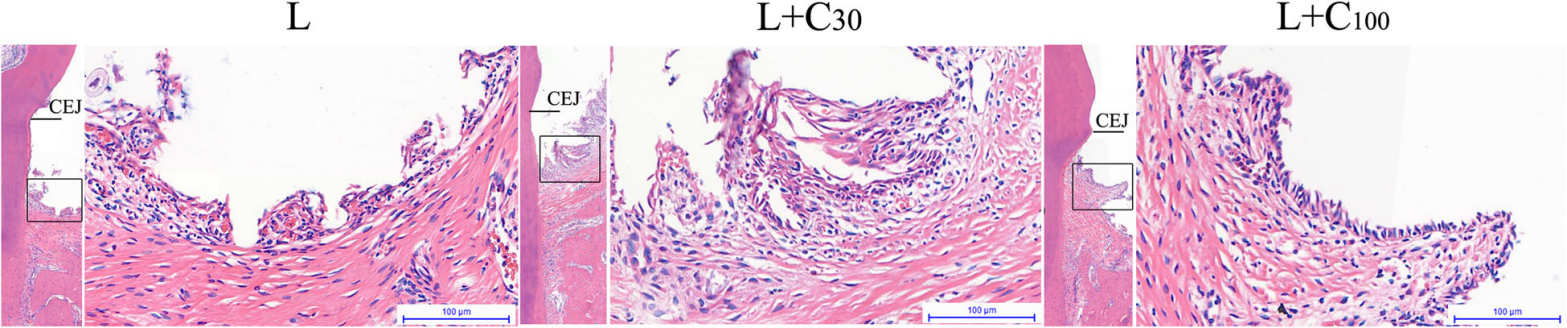
Histological analysis of gingival inflammation in rat experimental periodontitis. Gingival inflammation and bone loss were observed in all three groups. Alveolar bone crest height was obviously decreased in the ligation-only (L) group. A significant reduction in gingival inflammation and bone loss was observed in the groups that underwent ligation in combination with treatment with 30 μg/g body weight curcumin (L + C_30_) or 100 μg/g body weight curcumin (L + C_100_). CEJ, cementoenamel junction

**Fig. 8:**
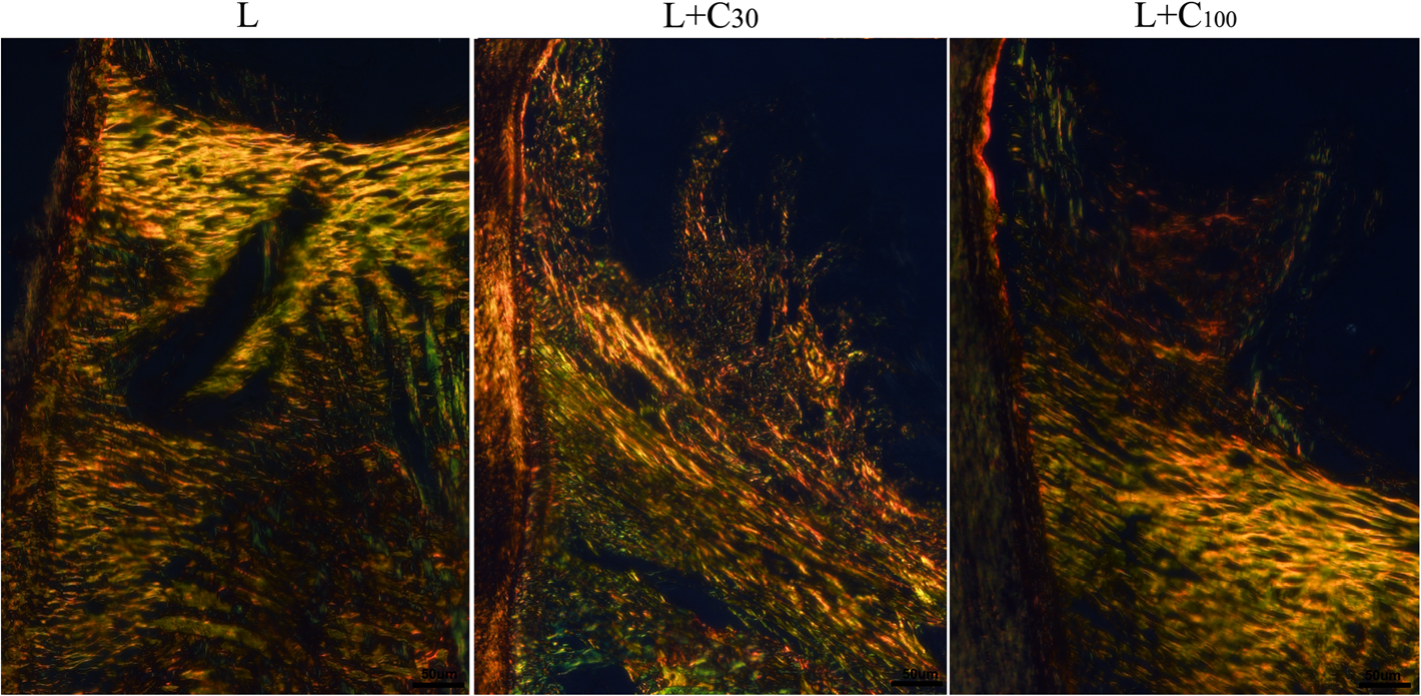
Picro-sirius red staining. Marked collagen fiber destruction was observed in the ligation-only (L) group. The fiber bundles were scattered and disordered. Collagen fiber destruction was alleviated in the groups that underwent ligation in combination with treatment with 30 μg/g body weight curcumin (L + C_30_) or 100 μg/g body weight curcumin (L + C_100_)

The histological changes in rat experimental periodontitis with the treatment of curcumin. Histological analysis revealed gingival inflammation in all three groups. However, a significant reduction in gingival inflammation and bone loss was observed in the L + C_30_ and L + C_100_ groups (Fig. 7). According to Picrosirius red staining, seriously collagen fiber destructions were observed in the L group. The fiber bundles were scattered and disordered. This collagen fiber destructions were also alleviated in the L + C_30_ and L + C_100_ groups (Fig. 8).

## Discussion

Curcumin has been demonstrated to have various biological properties, including anti-inflammatory, antioxidant, antimicrobial, and antiviral effects. Because of these properties, curcumin provides a very promising approach for the treatment of periodontitis [18, 19]. This study aimed to investigate the anti-inflammatory effects of curcumin on LPS-stimulated rat gingival fibroblasts and the underlying molecular mechanisms of these effects, which remain unclear. The cytotoxic effect of curcumin on rat gingival fibroblasts was assessed by the MTT assay in vitro. There was no significant difference between curcumin-treated fibroblasts and normal fibroblasts. So we did not use any negative or placebo controls in vivo for the consistency of experimental comparisons as we mainly aimed at the mechanism of curcumin in anti-inflammatory action.

TNF-α and IL-1β, as two of the important pro-inflammatory mediators, were significantly up-regulated in the process of periodontitis [20, 21], which are actively involved in jeopardizing periodontal tissues by affecting the activities of leukocytes, oteoclasts and collagenolytic enzyme MMPs to mediate alveolar bone resorption and collagen destruction [22, 23]. So, in this study we chose TNF-α and IL-1β to examine the effect of curcumin on the production of these cytokines, since these cytokines participate to various extent in the production and the development of inflammation through recruitmnt and activation of inflammatory cells [24].

Gingival tissues were first invaded and stimulated by periodontal bacteria and their metabolic products in the initial process of periodontitis. The overproduction of IL-1β and TNF-α have been known to play important roles in periodontal inflammatory degradation [25]. According to our ELLSA results, curcumin inhibited the production of IL-1β and TNF-α in rat gingival fibroblasts induced by LPS, which showed that curcumin has potential role in modulating immune response associated with periodontal diseases.

To investigate the inflammatory mechanism, the effects of curcumin on LPS-induced NF-κB activation were detected by western blotting. Results showed that curcumin significantly inhibited upregulated NF-κB p65 and IκB phosphorylation induced by LPS.

NF-κB activation can stimulate a number of inflammatory events and amplify the inflammatory responses, including inducing adhesion molecules, and activating matrix metalloproteinase, which occur in periodontal disease process. NF-κB activation in gingival fibroblasts leads to the over-release of proinflammatory cytokines IL-1β and TNF-α, which further enhanced periodontal tissue destruction. IL-1 and TNF-α also promote the recruitment and activity of osteoclasts, by enhancing production of a crucial osteoclastogenic factor, the Receptor Activator of Nuclear Factor κ B Ligand (RANKL) and favor bone destruction [26].

The expression/activation of OPG and RANKL are crucial for alveolar bone absorption and metabolism [27, 28]. The osteoclast differentiates from monocyte/macrophage precursors under the regulation of RANKL/RANK signaling. OPG is a secreted protein that protects bone from excessive resorption by binding to RANKL and preventing it from binding to RANK [29–31]. Soluble RANKL (sRANKL) and OPG from gingival fibroblasts stimulated by LPS may interrupt alveolar bone metabolism by paracrine secretion. Thus, OPG/sRANKL ratio is a major determinant. According to our results, OPG/sRANKL ratio in culture supernatants of gingival fibroblasts was decreased when incubated with LPS, curcumin alleviated LPS-induced down-regulated OPG/sRANKL ratio. Curcumin may alleviate LPS-induced osteoclast activation and alveolar bone absorption by down-regulating OPG/sRANKL ratio.

In vivo, histological observation and micro-CT results showed gingival inflammation and alveolar bone loss was observed in rat experimental periodontitis. Both 30 and 100 μg / g / body weight of curcumin could alleviate the gingival inflammation and alveolar bone loss [32–35]. According to Picrosirius red staining, the fiber bundles became scattered and disordered in rat experimental periodontitis. Collagen fiber destructions were also alleviated by curcumin.

## Conclusion

In the present study, we provided new evidence on the inhibitory effect of curcumin on inflammatory activity. Curcumin significantly reduced gingival inflammation and modulated collagen fiber and alveolar bone loss in vivo. Curcumin can significantly inhibit NF-κB activation and decrease the OPG/sRANKL ratio induced by LPS. This study provides a new anti-inflammatory therapeutic for periodontal diseases.
